# Dysfunction in Ribosomal Gene Expression in the Hypothalamus and Hippocampus following Chronic Social Defeat Stress in Male Mice as Revealed by RNA-Seq

**DOI:** 10.1155/2016/3289187

**Published:** 2015-12-29

**Authors:** Dmitry A. Smagin, Irina L. Kovalenko, Anna G. Galyamina, Anatoly O. Bragin, Yuriy L. Orlov, Natalia N. Kudryavtseva

**Affiliations:** ^1^Modeling of Neuropathology Laboratory, Institute of Cytology and Genetics, Siberian Department of Russian Academy of Sciences, Novosibirsk 630090, Russia; ^2^Laboratory of Behavioral Neuroinformatics, Institute of Cytology and Genetics, Siberian Department of Russian Academy of Sciences, Novosibirsk 630090, Russia; ^3^Novosibirsk State University, Novosibirsk 630090, Russia

## Abstract

Chronic social defeat stress leads to the development of anxiety- and depression-like states in male mice and is accompanied by numerous molecular changes in brain. The influence of 21-day period of social stress on ribosomal gene expression in five brain regions was studied using the RNA-Seq database. Most *Rps, Rpl, Mprs*, and *Mprl* genes were upregulated in the hypothalamus and downregulated in the hippocampus, which may indicate ribosomal dysfunction following chronic social defeat stress. There were no differentially expressed ribosomal genes in the ventral tegmental area, midbrain raphe nuclei, or striatum. This approach may be used to identify a pharmacological treatment of ribosome biogenesis abnormalities in the brain of patients with “ribosomopathies.”

## 1. Introduction

Chronic social defeat stress (CSDS) can lead to the development of behavioral psychopathology, which is accompanied by anxiety- and depression-like states in male mice [1–3] similar to those in humans. It has been shown that, under CSDS, the adult brain undergoes numerous changes, including changes in gene expression in different brain regions [3, 4], DNA methylation, histone acetylation, and chromatin remodeling [5, 6] as well as decreases in hippocampal neurogenesis [7–9].

Analyzing the whole transcriptome using RNA-Seq in five brain regions of depressive mice with chronic social defeats experience, we observed changes in the expression of numerous genes. This report is concentrated on the analysis of genes encoding ribosomal and mitochondrial ribosomal proteins (*Rps* and* Rpl, Mrpl* and* Mrps*) which are responsible for translation, transcription, and proliferation and are involved in neural plasticity in healthy cells.

Two groups of animals were analyzed: male mice in a depression-like state following CSDS over a 21-day period of agonistic interactions and control mice. Choice of the brain regions selected for testing was based on their functions, localization of neurons of some neurotransmitter systems, and differential involvement in the mechanisms of a depression-like state in our experimental paradigm [1, 10]. These regions are as follows: the midbrain raphe nuclei, a multifunctional brain region, which contains the majority of serotonergic neuronal bodies; the ventral tegmental area (VTA), which contains the bodies of dopaminergic neurons, is widely implicated in natural reward circuitry of the brain, and is important in cognition, motivation, drug addiction, and emotions relating to several psychiatric disorders; the striatum, which is responsible for the regulation of motor activity and stereotypical behaviors and is also potentially involved in a variety of cognitive processes; the hippocampus, which belongs to the limbic system, is essential for memory consolidation and storage, and plays important roles in the neurogenesis and emotional mechanisms; and the hypothalamus, which regulates the stress reaction and many other physiological processes.

## 2. Materials and Methods

### 2.1. Animals

Adult male mice of C57BL/6J were obtained from Animal Breeding Center in Pushchino (Moscow region, Russia). Animals were housed under standard conditions (12:12 hr light/dark regime; switch-on at 8.00 a.m.: at temperature of 22 ± 1°C; and food (pellets) and water available* ad libitum*). Experiments were performed on 10–12-week-old animals. All procedures were in accordance with the European Communities Council Directive of November 24, 1986 (86/609/EEC). The study was approved by Scientific Council number 9 of the Institute of Cytology and Genetics SB RAS of March, 24, 2010, N 613.

### 2.2. Chronic Social Defeat Stress

Prolonged negative social experiences (defeats) in male mice were induced by daily agonistic interactions with an aggressive partner [1, 10]. Pairs of weight-matched animals were placed in steel cages (14 × 28 × 10 cm) bisected by a perforated transparent partition, which allowed the animals to see, hear, and smell each other but prevented physical contact. The animals were left undisturbed for two days to allow for adaptation to the new housing conditions and sensory contact before they were exposed to encounters. Every afternoon (14:00–17:00 p.m. local time), a transparent cage lid was placed on the cage, and, 5 min later (the period necessary for individual activation), the partition was removed for 10 minutes to encourage agonistic interactions. The superiority of one of the mice was firmly established within two or three encounters with the same opponent. The winning mouse would attack, bite, and chase the losing mouse, which would display only defensive behavior (sideways posture, upright posture, withdrawal, lying on the back, or freezing). As a rule, aggressive confrontations between males were discontinued by lowering the partition if the sustained attacks had lasted 3 min or less to prevent damage to the losers. Each defeated mouse (loser or defeater) was exposed to the same winner for three days; afterwards, each loser was placed, once a day after the fight, in an unfamiliar cage with an unfamiliar winner behind the partition. Each winning mouse remained in its original cage. This procedure was performed for 21 days and yielded an equal number of winners and losers. Two groups of animals were analyzed in this experiment: (1) depressive mice: groups of chronically defeated mice on 21st days of agonistic interactions and (2) controls: the mice without any consecutive experience of agonistic interactions. The detailed description of this behavioral method has been previously published [10].

All of the mice were decapitated simultaneously: the 21-time defeated mice were sacrificed 24 hours after the last agonistic interaction and the control animals. The brain regions from both experimental groups were dissected by one experimenter according to the map presented in the Allen Mouse Brain Atlas (http://mouse.brain-map.org/static/atlas). All of the biological samples were placed to the RNAlater solution (Life Technologies, USA) and stored at −70°C until sequencing.

### 2.3. RNA-Seq Method

The collected samples were sequenced at JSC Genoanalytica (http://genoanalytica.ru/, Moscow, Russia), where the mRNA was extracted using the Dynabeads mRNA Purification Kit (Ambion, USA). cDNA libraries were constructed using NEBNext mRNA Library PrepReagent Set for Illumina (NEB, USA) following the manufacturer's protocol and were subjected to Illumina sequencing. More than 20 million reads were obtained for each sample. The resulting “fastq” format files were used to align all of the reads to the GRCm38.p3 reference genome using the TopHat aligner [11]. The Cufflinks program was used to estimate the gene expression levels in FPKM (fragments per kilobase of transcript per million mapped reads) and then to detect the differentially expressed genes (DEGs) in the analyzed and control groups. Each brain region was considered separately for 3 versus 3 animals. Only annotated gene sequences were used in the following analysis. Genes were considered to be differentially expressed at *P* < 0.01.

## 3. Results and Discussion

Gene expression levels were compared between mouse groups affected by social stress, depressive mice, and the control animals. Analysis of differentially expressed genes showed their dependence on the brain regions (Table 1): in the hypothalamus, 3703 genes changed their expression pattern under CSDS (ratio of up/down is 2244/1459, resp.); in the striatum, 931 genes changed their expression pattern under CSDS (up/down, 770/161); in the hippocampus, 841 genes changed their expression pattern under CSDS (up/down, 423/418); in the VTA, 549 genes changed their expression pattern under CSDS (up/down, 229/320); and, in the raphe nuclei, 453 genes changed their expression pattern under CSDS (up/down, 104/349) at the chosen level of statistical significance (*P* < 0.01). Thus, the largest number of differentially expressed genes in depressive mice was observed in the hypothalamus, and approximately 4 times fewer genes were observed in the striatum and hippocampus and approximately 7 times fewer genes were observed in the VTA and midbrain raphe nuclei. In the hypothalamus and striatum, the number of upregulated genes was higher than number of downregulated genes. In the VTA and midbrain raphe nuclei area, most of the differentially expressed genes were downregulated. In the hippocampus, the numbers of upregulated and downregulated genes were approximately equal. We can assume that the number of differentially expressed genes and direction of change (up or down) may be used as marker of more or less intensive involvement of any brain area into molecular mechanisms of depression-like state in mice. These changes may depend on function of brain regions and different mechanisms regulating CSDS. Other authors have demonstrated changes of numerous genes expression in the nucleus accumbens under CSDS [3].

In the midbrain raphe nuclei, VTA, and striatum, the* Rps, Rpl, Mrpl,* and* Mrps* genes did not change their expression under CSDS. Because ribosomes are responsible for protein synthesis in all cells, we suspect that the translation, transcription, and proliferation of proteins are not significantly disturbed in these brain regions.

In the hippocampus and hypothalamus, the major components of ribosomes—the small ribosomal subunit that reads the RNA (*Rps*) and the large subunit that connects amino acids to form a polypeptide chain (*Rpl*)—changed their expression under CSDS. In the hippocampus of depressive mice, the largest number of ribosomal genes (*Rpl7, Rpl36a, Rpl39, Rps4x,* and* Rps27a*) was downregulated and only 2 genes (*Rpl35* and* Rpl18*) were upregulated. We can assume that downregulation of the ribosomal genes may be associated with a decrease of proliferation in the hippocampal dentate gyrus under CSDS in mice, as described by many authors using a similar experimental paradigm [7–9].

**Figure 1:**
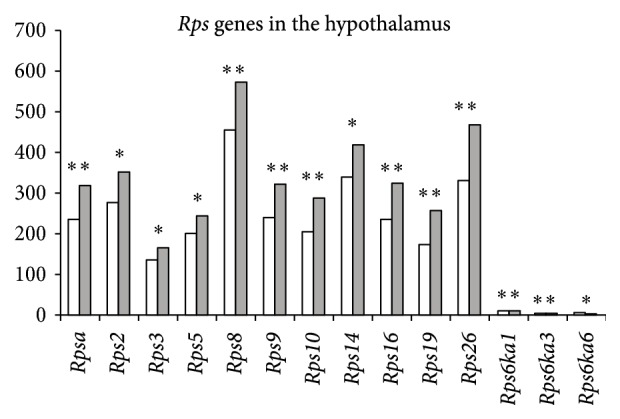
The differentially expressed ribosomal* Rps* genes in the hypothalamus of mice following CSDS. The Cufflinks program was used to estimate the gene expression levels in FPKM. The levels of the* Rps* gene expression are presented in the control (left columns) and depressive mice (right columns). The* Rps14, Rps8, Rps6ka1, Rps9, Rps5, Rps19, Rps16, Rps3, Rpsa, Rps2, Rps26,* and* Rps10* genes were upregulated, whereas* Rps6ka3* and* Rps6ka6* were downregulated under CSDS in depressive mice. Statistical significance *P* < 0.01 and *q* < 0.05. ^*∗*^ *P* < 0.01; ^*∗∗*^ *P* < 0.001.

**Figure 2:**
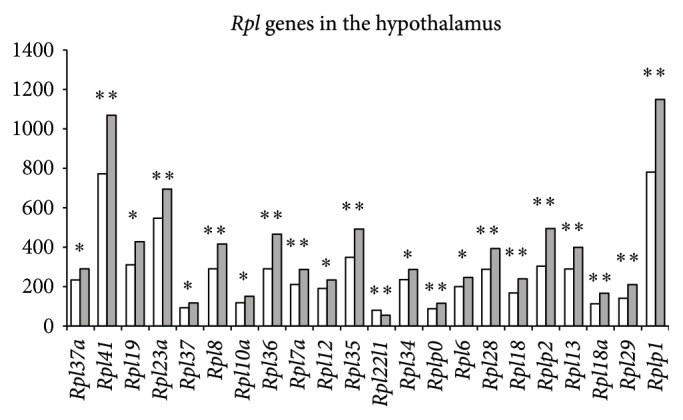
Differentially expressed ribosomal* Rpl* genes in the hypothalamus of mice following CSDS. The Cufflinks program was used to estimate the gene expression levels in FPKM. The levels of the* Rpl* genes expression are presented in the control (left columns) and depressive mice (right columns). The* Rpl37a, Rpl41, Rpl19, Rpl23a, Rpl37, Rpl8, Rpl10a, Rpl36, Rpl7a, Rpl12, Rpl35, Rpl34, Rplp0, Rpl6, Rpl28, Rpl18, Rplp2, Rpl13, Rpl18a, Rpl29,* and* Rplp1* genes were upregulated, whereas the* Rpl22l1* gene was downregulated. Statistical significance *P* < 0.01 and *q* < 0.05. ^*∗*^ *P* < 0.01; ^*∗∗*^ *P* < 0.001.

In the hypothalamus, numerous ribosomal genes changed their expression under CSDS (14* Rps* and 22* Rpl* genes).* Rps14, Rps8, Rps6ka1, Rps9, Rps5, Rps19, Rps16, Rps3, Rpsa, Rps2, Rps26, *and* Rps10* and* Rpl37a, Rpl41, Rpl19, Rpl23a, Rpl37, Rpl8, Rpl10a, Rpl36, Rpl7a, Rpl12, Rpl35, Rpl34, Rplp0, Rpl6, Rpl28, Rpl18, Rplp2, Rpl13, Rpl18a, Rpl29,* and* Rplp1* were upregulated, and* Rpl22l1, Rps6ka3,* and* Rps6ka6* were downregulated (Figures 1 and 2). Enhanced expression of the* Rpl18* and* Rpl35* genes was overlapped in the hippocampus and hypothalamus.

The hypothalamus is responsible for production of numerous hormones that are involved in the regulation of many physiological functions and psychoemotional states. Many diseases are connected with abnormal hypothalamic function, such as changed stress reactions, metabolism, loss or increase of appetite, changed emotional behavior, memory loss, sleep disorders, and affective and somatic states. Because decreased stress reactivity, weight loss, and development of pronounced anxiety- and depression-like state were observed in the mice after CSDS [1, 12], we suggest a significant involvement of the hypothalamus in these pathological processes. Support for this hypothesis comes from the observation that the largest number of all differentially expressed genes was observed in this region. The majority of these genes (60%), including ribosomal genes, were upregulated in depressive mice. However, does upregulation of numerous ribosomal genes present a feedback mechanism in response to hypothalamic activation under CSDS, or is this a result of ribosomal gene dysfunction developing in depressive mice?

In recent years a number of human diseases have been identified and categorized as “ribosomopathies” [13, 14] caused by alterations in either the structure or function of ribosomal components, which are associated with distinct mutations in the ribosomal biogenesis pathway. These diseases include Diamond-Blackfan anemia, Shwachman-Diamond syndrome, and dyskeratosis congenita. The* Rps10* and* Rps26* genes are commonly mutated in Diamond-Blackfan anemia and have been associated with mutations in seven other ribosomal protein genes (*Rps19, Rps24, Rps17, Rpl35A, Rpl5, Rpl11*, and* Rps7*) in approximately 43% of patients [15, 16]. Interestingly, increased expression of the* Rps19, Rps14, Rps10,* and* Rps26* genes, which are involved in Diamond-Blackfan anemia, was observed in depressive mice. We did not find literature data concerning ribosome dysfunction during depression; however, our observation concerning changes in the expression of ribosomal genes in the hippocampus and hypothalamus in mice indicates developing ribosomal dysfunction under CSDS.

**Figure 3:**
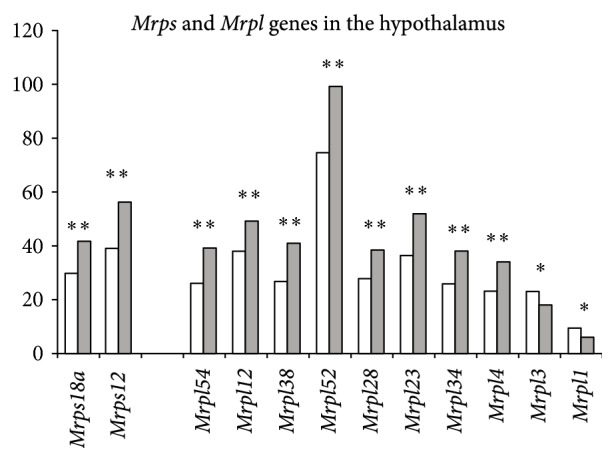
Differentially expressed mitochondrial ribosomal* Mrps* and* Mrpl* genes in the hypothalamus of mice following CSDS. The Cufflinks program was used to estimate the gene expression levels in FPKM. The levels of the* Mrps* and* Mrpl* gene expression are presented in the control (left columns) and depressive mice (right columns). The* Mrpl54, Mrpl12, Mrpl38, Mrpl52, Mrpl28, Mrpl23, Mrpl34, Mrpl4, Mrps18a, Mrps12, Mrps18a,* and* Mrps12* genes were upregulated, whereas* Mrpl1 and Mrpl3* were downregulated. Statistical significance *P* < 0.01 and *q* < 0.05. ^*∗*^ *P* < 0.01; ^*∗∗*^ *P* < 0.001.

There were no mitochondrial ribosomal genes found that changed expression under CSDS in the hippocampus. However, in the hypothalamus the results obtained indicate the development of possible mitochondrial protein dysfunctions: the mitochondrial ribosome genes* Mrpl54, Mrpl12, Mrpl38, Mrpl52, Mrpl28, Mrpl23, Mrpl34, Mrpl4 Mrps18a,* and* Mrps12* were upregulated in depressive mice, whereas* Mrpl1* and* Mrpl3* were downregulated (Figure 3). Thus, we can assume a strong link between CSDS leading to the development of a depression-like state in mice and the activation of mitochondrial ribosomal genes in the hypothalamus. These suppositions are confirmed indirectly by experimental data that have demonstrated the upregulation of mitochondrial genes in the amygdala of rats in a depression-like state induced by inescapable tail shock [17]. In a genetic model of depression, changes in the number and morphology of mitochondria in the hippocampus were shown [18]. Another author group observed the influence of chronic unpredictable stress on the serotonin levels in the raphe nuclei and hippocampus and overactivation of mitochondria in the raphe nuclei of mice [19]. Earlier we found decreased brain serotonergic activity in depressive mice as shown by decreased serotonin levels and/or 5-hydroxyindoleacetic acid and tryptophan hydroxylase activity, the key limiting enzyme of serotonin synthesis, in different brain areas [2, 20], as well as downregulation of serotonergic* Tph2, Sert, Maoa,* and* Htr1a* gene expression, which are associated with the synthesis, inactivation, and reception of serotonin, respectively, in the midbrain raphe nuclei [21]. We can suggest that overactivation of mitochondria, determined by the respiratory control ratio, ATP synthesis rate, and activities of superoxide dismutase and glutathione peroxidase shown by authors [19] in the raphe nuclei may be result of feedback mechanisms on the development of hypofunction of serotonergic activity [2, 20, 21] in this brain region of stressed mice. Conversely, in the hypothalamus, activation of tryptophan hydroxylase was observed in depressive mice [2, 20]. It could be assumed that the development of mitochondrial dysfunction in depressive mice is associated with activation of serotonergic system, at least in the hypothalamus. This conclusion is indirectly confirmed by observations that patients with mitochondrial disorders can show primary psychiatric symptomatology, including mood disorder, cognitive impairment, psychosis, and anxiety [22].

Mitochondrial disorders may be caused by either acquired or inherited mutations in the mitochondrial DNA or in nuclear genes that code for mitochondrial components [23]. These disorders may also be the result of acquired mitochondrial dysfunction due to adverse effects of drugs, infections, or other environmental causes. The majority of mitochondrial disorders are associated with neurological abnormalities, including seizures and myoclonus, psychomotor retardation, dementia, ataxia, motor neuron disease, weakness, and chronic fatigue [24]. Depressive mice have been shown to demonstrate also motor retardation, immobility, and helplessness in any situations [1, 2, 12].

Undoubtedly it is difficult to find a direct association between the overexpression of ribosomal genes and mitochondrial ribosomal genes in the hypothalamus and the depression-like state in mice, which would help to understand causes and consequences of these processes. At this stage of research, it is impossible to elucidate the detailed sequence of neurochemical events involved, and, as a result, the molecular changes that occur due to restructuring brain regulation in male mice under CSDS. However, it is clear that, starting with a change in social behavior and psychoemotional state under CSDS, at certain stages this process launches a cascade of systemic changes at the whole brain level, its regions, and specific neurons following changes in metabolism and reception of neurotransmitter systems. As a result, it leads to the changes in the expression of genes involved in the development of affective disorders. The changes observed in ribosomal and mitochondrial ribosomal gene expression may indicate ribosome dysfunction. Our model, which induces a mixed anxiety/depression-like state [1, 2] in male mice following CSDS may be used to identify a pharmacological treatment of ribosome biogenesis abnormalities in the brain.

## Figures and Tables

**Table 1:** The number of genes that changed their expression in brain regions of depressive male mice.

	Raphe nuclei	Hippocampus	VTA	Striatum	Hypothalamus
All genes	453	841	549	931	3703
Upregulated	104	423	229	770	2244
Downregulated	349	418	320	161	1459

Ribosome genes	Up/down	Up/down	Up/down	Up/down	Up/down

*RPS *	0	0/2	0	0	13/2
*RPl *	0	2/3	0	0	21/1
*Mrps *	0	0	0	0	2/0
*Mrpl *	0	0	0	0	8/2
